# Innovations in Glaucoma Surgery: Improving the Results

**DOI:** 10.1155/2016/5856075

**Published:** 2016-07-21

**Authors:** Michele Figus, Shlomo Melamed, Antonio Ferreras, Giorgio Marchini, Vital P. Costa

**Affiliations:** ^1^University of Pisa, 56126 Pisa, Italy; ^2^University of Tel Aviv, 6997801 Tel Aviv, Israel; ^3^Miguel Servet University of Zaragoza, 50009 Zaragoza, Spain; ^4^University of Verona, 37129 Verona, Italy; ^5^University of Campinas, 13083-970 Campinas, SP, Brazil

Glaucoma still represents the most frequent cause of irreversible blindness worldwide. The technological advances lead to innovative surgeries, which are rapidly introduced in clinical practice. New devices to lower intraocular pressure without opening the eye wall, bypassing the trabecular meshwork, or shunting the aqueous humor to the suprachoroidal space have been approved undergoing clinical assessment within clinical trials. Currently, other devices under evaluation are showing promising results. Although these methods will increase the options available for glaucoma surgeons, it is unclear if they could replace the present standard surgeries, such as trabeculectomy, deep sclerectomy, and glaucoma drainage devices. Moreover, the standard procedures are continuously modified in different ways to become safer and more effective.

In this issue L. Choritz et al. investigate whether increased concentrations of endothelin-1 in the aqueous humor samples of glaucoma patients influenced wound healing and bleb fibrosis after standard trabeculectomy with mitomycin C. Endothelin-1 is a potent vasoconstrictor produced in the eye by the ciliary epithelium and to be released into the aqueous humor. It has been implicated in the pathophysiology of glaucoma. Endothelin-1 is believed to be involved in the regulation of intraocular pressure (IOP) via effects on the contractility of ciliary muscle and trabecular meshwork.

W. Niu et al. evaluated the efficacy and safety of three different biodegradable terpolymers after trabeculectomy in rabbit eyes compared with Ologen*™*. The use of these implants aims to reduce the use of mitomycin C as an antiscarring agent. The first implant used was a porous collagen-glycosaminoglycan matrix, which prevents the adhesion of the conjunctiva and sclera and the collapse of the subconjunctival space after trabeculectomy, leading to collagen deposition and microcyst formation after penetrating antiglaucomatous surgery.

Despite the increasing use of antifibrotic agents to modulate the wound healing response, bleb failure remains a common complication of glaucoma filtration surgery. In the paper by W. Liu et al. the needle revision and subconjunctival mitomycin C injection were compared with needling and subconjunctival 5-Fluorouracil injection for early dysfunctional filtration blebs after trabeculectomies.

Neovascular glaucoma is one of the most recalcitrant glaucoma types to treatment and has one of the worst outcomes compared to other types of glaucoma. Neovascular glaucoma often needs surgical treatment because medical treatment fails to adequately control intraocular pressure. In a retrospective study, H. Yan investigated the long term surgical outcomes, treating neovascular glaucoma complicated by vitreous haemorrhage with 23-gauge vitrectomy combined with phacoemulsification, panretinal laser photocoagulation, and trabeculectomy without using anti-VEGF agents.

C. Cagini et al. explored the role of canaloplasty in the surgical management of glaucoma with an extensive review. Canaloplasty is a nonpenetrating blebless surgical technique for open-angle glaucoma, similar to viscocanalostomy, in which a flexible microcatheter is inserted within Schlemm's canal for the entire 360 degrees. The results of this ab externo technique are really encouraging. S. A. Gandolfi et al. compared canaloplasty with an ab interno minimally invasive glaucoma surgery, the Hydrus*™* implant. Both techniques are innovative and aim to restore the natural pathway, dilating a large size of Schlemm's canal. The results of this retrospective, comparative case series were collected after 2-year follow-up.

A. F. Resende et al. presented a review on the iStent. This trabecular bypass stent is another minimally invasive glaucoma surgery device that has quickly gained popularity in the last years. Randomized controlled trials are still needed to assess the role of these devices in glaucoma surgery.

Finally, S. Jacob et al. presented a new surgical technique, the stab incision glaucoma surgery. Even though it looks similar to standard trabeculectomy the main advantages are the sliding of the superior conjunctiva without dissecting, the creation of a superficial corneoscleral tunnel in a single step, the punch of the internal lip of the tunnel, and finally the suture of the conjunctival incision alone.

In summary, this issue includes different approaches presented by diverse authors covering several topics related to improving of standard filtering surgery and wound healing and to new surgical techniques. This publication will provide valuable information that should be helpful in clinical practice for the whole ophthalmology community, mainly for glaucoma surgeons.

*Michele Figus* *Michele Figus* *Shlomo Melamed* *Shlomo Melamed* *Antonio Ferreras* *Antonio Ferreras* *Giorgio Marchini* *Giorgio Marchini* *Vital P. Costa* *Vital P. Costa*

